# Amantadine-associated delirium in patients with maintenance dialysis: Insomnia-associated recovery and uneven seasonal distribution

**DOI:** 10.1097/MD.0000000000034077

**Published:** 2023-06-30

**Authors:** Jing Li, Bolin Si, Jun Chao, Jianqiang He

**Affiliations:** a Department of Nephrology, Affiliated Hospital of Jiangsu University, Zhenjiang, China.

**Keywords:** amantadine hydrochloride, delirium, dialysis, kidney, sleep disorders, survival

## Abstract

Amantadine hydrochloride is a risky drug for triggering delirium in dialysis patients; however, it is often administered casually. Furthermore, little is known regarding the recovery and prognosis of dialysis patients with amantadine-associated delirium. Data of this retrospective cohort study were collected from a local hospital database for hospitalizations between January 2011 and December 2020. Patients were divided into 2 cohorts: early recovery (recovery within 14 days) and delayed recovery (recovery more than 14 days). The cases were analyzed together with the intermonth temperature using descriptive statistics. A Kaplan–Meier survival curve and binary logistic regression were applied for the analyses of prognoses and factors. A total of 57 patients were included in this study. The most common symptoms were hallucinations (45.61%) and muscle tremors (43.86%). Early recovery was observed in 63.16% of the patients. Only 3.51% of the cases occurred in local summer (June, July, and August). Better prognoses for survival (hazard ratio [HR] = 0.066, 95% confidence interval [95% CI] = 0.021–0.212) and hospitalization costs (7968.42 ± 3438.43 CNY vs 12852.38 ± 9361.13 CNY, *P* = .031) were observed in patients with early recovery than in those with delayed recovery. In the multivariate logistic regression adjusted by 1:1 propensity score matching, delayed recovery was independently caused by insomnia (*P* = .022, $\hat{OR}$ = 10.119, 95% CI = 1.403–72.990) and avoided in patients with urine volume over 300 mL (*P* = .029, $\hat{OR}$ = 0.018, 95% CI = 0.006–0.621). The increment (per 100 mg) of cumulative dose (*P* = .190, $\hat{OR}$ = 1.588, 95% CI = 0.395–3.172) tended to be a risk of delayed recovery. The area under curve of the receiver operating characteristic curve was 0.867, with a sensitivity of 90.5% and a specificity of 82.4% at the cutoff point (cutoff = 0.432). For amantadine-associated delirium in dialysis patients with uneven seasonal distribution, early recovery with better prognosis should be the aim of treatment by giving priority to the remedy of insomnia.

## 1. Introduction

As the main treatment for irreversible chronic kidney failure, dialysis has spread worldwide during the past 2 decades owing to the increasing prevalence of end-stage renal disease (ESRD).^[1]^ As a result, emerging dialysis-related diseases and complications have been recognized as important mortality causes and aggravated burdens for both patients and the society.^[2,3]^ Mental disorders are common yet undervalued problems that cause mortality and greatly diminish the quality of life of patients.^[4,5]^ Approximately 2% of adults and 1% of children with ESRD are hospitalized with a primary psychiatric diagnosis.^[6]^ Patients undergoing maintenance dialysis are much more susceptible to the cumulative toxicity of inappropriate drug administration due to the loss of kidney function,^[7]^ which is commonly found in the administration of antibiotics.^[7]^

Amantadine hydrochloride is generally used to treat influenza A viral infections. It has recently been proven effective for coronavirus disease-19 treatment.^[8,9]^ It is a noncompetitive N-methyl-D-aspartate receptor antagonist and is approved for the treatment of drug-induced dyskinesia caused by levodopa in patients with Parkinson’s disease^[10]^{Marmol, 2021 #82}{Marmol, 2021 #82}. Amantadine hydrochloride is mainly metabolized and excreted by the kidneys. Moreover, it is not fully removed via a single dialysis session,^[11]^ making its use even riskier for patients with ESRD. Case reports of amantadine-induced delirium or coma in patients with renal deficiency were published in 1974 and 1993.^[12,13]^ However, in China, amantadine is commonly used as the main ingredient in over-the-counter medicines for treating colds.^[14]^ These over-the-counter medicines can be easily purchased and taken without any further consultation, even for patients on maintenance dialysis.

Despite the emerging cases of amantadine-associated delirium,^[13–16]^ few studies have been conducted to understand the demographics or comorbidities of dialysis patients with amantadine-associated delirium, as well as the characteristics for their occurrence and recovery. This is partly due to the sporadic occurrence and reversibility of symptoms in the majority of such cases.^[16]^ This study aimed to present a general outline of the clinical characteristics of hospitalized dialysis patients with delirium after amantadine administration. Additionally, we investigated how the duration of delirium could affect the prognoses of those patients on maintenance dialysis and what the influential factors for recovery were.

## 2. Material and methods

### 2.1. Subjects

Data from patients on maintenance dialysis hospitalized due to newly occurring delirium after amantadine administration in the Department of Nephrology, Jiangsu University Affiliated Hospital from January 2011 to December 2020 were collected retrospectively with the following inclusion and exclusion criteria.

Inclusion criteria included the following:

1. maintenance dialysis patients (≥3 months) hospitalized in the Department of Nephrology, Jiangsu University Affiliated Hospital from January 2011 to December 2020,
2. recent (within 3 days) amantadine hydrochloride administration before the onset of delirium, and
3. aged between 18 and 80 years.

Exclusion criteria included the following:

1. patients with irregular dialysis,
2. patients with recurrent mental symptoms in the past 1 year,
3. patients detected with a mass or newly occurred cerebrovascular lesions,
4. patients with decompensated hepatocirrhosis,
5. patients with respiratory failure requiring mechanical ventilation, and
6. patients with severe electrolyte disorders.

### 2.2. Data collection

This was a retrospective cohort study, and the following patient data were collected from the hospital database: sex, age, primary cause for ESRD, insomnia, antibiotic administration, cumulated dose, and duration of amantadine administration, comorbidities (hypertension, diabetes, coronary heart disease, chronic obstructive pulmonary disease, and hepatitis), dialysis treatment (type, frequency, and dialysis age), medication administration, general status at the onset of delirium (time and symptoms), laboratory tests (levels of serum creatine, serum urea nitrogen, hemoglobin, alanine aminotransferase, aspartate aminotransferase, albumin, total bilirubin, parathyroid hormone, serum phosphate, C-reactive protein, triglycerides, and total cholesterol), treatments in the hospital (hemoperfusion, olanzapine, quetiapine, and haloperidol), duration of recovery, costs for hospitalization, etc.

### 2.3. Cohorts and prognosis data

Since the delirium is mostly reversible according to previous reports,^[12–16]^ the duration of recovery and costs for hospitalization were considered important outcomes. Considering the pharmacokinetics of amantadine and the average length of stay,^[11,17]^ recovery within 14 days was considered early recovery. Accordingly, the patients were divided into 2 cohorts: early recovery (recovery within 14 days) and delayed recovery (recovery more than 14 days). For further prognosis, retrospective data concerning death and readmission were collected until December 2021 for all patients after discharge, along with the duration of survival (counted in months).

### 2.4. Insomnia assessment

The sleeping status of patients was evaluated via the Athens insomnia scale^[18]^ based on the evaluation on the first day of hospitalization. Patients with over 10 scores were considered to have insomnia.

### 2.5. Temperature data

Monthly temperature data of the local area over the past 10 years from 2011 to 2021 were obtained from the website of National Centers for Environmental Information.^[19]^ Average data (both minimum and maximum temperatures) for the same month in different years were statistically analyzed to present a trend of seasonal changes.

### 2.6. Statistical analyses

Statistical analyses were conducted using the SPSS 22.0, GraphPad Prism 8.0.1, and MedCalc 12. Mean ± standard deviation is used to describe numerical variables, and percentages are used to describe categorical variables. Student *t* test and Chi-square test (or Fisher’s exact test) were performed to compare variables between the 2 groups. A Kaplan–Meier survival curve was used for survival analysis, and Log-rank test was applied for comparison. For further comparison of influential factors, a propensity score matching (1:1) through SPSS was conducted to adjust for other potential factors causing delirium, including antibiotic administration, C-reactive protein, and serum albumin levels. Binary logistic regression analyses (with the methods of “Forward: LR” and “Enter”) were used to analyze factors influencing the duration of recovery. The sample size of multivariate logistic regression was evaluated by including covariates selected from the univariate logistic regression, and the sample size was approximately 10 to 20 times the number of covariates. *P* < .05 was considered statistically significant.

## 3. Results

### 3.1. Clinical characteristics

This study included retrospective data from 57 patients. All patients took amantadine because of a previous respiratory tract infection. More patients (66.67%) were male, and the average age was 63.54 ± 12.35 years. No patient died in the hospital, but 14 patients died during the follow-up. Early recovery was observed in 63.16% of the patients. The most common symptoms for patients in this study were hallucinations (45.61%), muscle tremor (43.86%), and hyperactivity (38.60%).

The average dialysis age was 2.43 ± 1.84 years. Most patients (85.96%) underwent hemodialysis, and the rest underwent peritoneal dialysis. The top 3 primary causes of ESRD were chronic nephritis (40.35%), diabetic nephropathy (29.82%), and hypertensive renal damage (22.81%).

Between the 2 group of patients observed with different recovery, there were no differences for most factors collected, especially for infection and nutrition related items, including antibiotic administration, C-reactive protein, and albumin (Table S1, Supplemental Digital Content, http://links.lww.com/MD/J164). Only insomnia (*P* = .001), urine volume > 300 mL (*P* = .001), and dialysis age < 1 year (*P* = .021) were statistically different between the 2 groups.

### 3.2. Seasonal variation of onset of delirium

**Figure 1.**
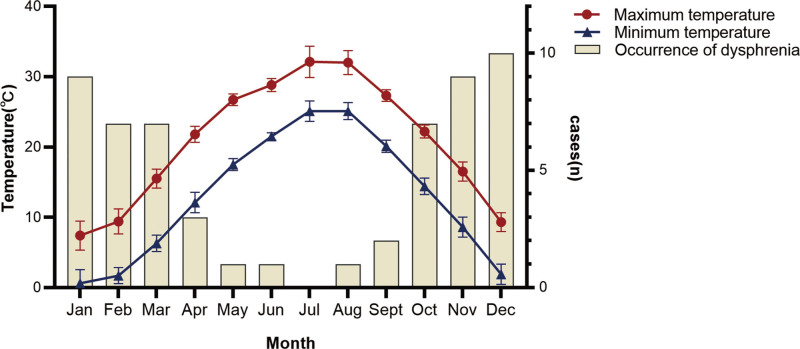
Seasonal variation for occurrence of delirium and the local temperature.

Regarding the onset of amantadine-associated delirium in all patients (Fig. 1), an uneven distribution was observed among different seasons from 2011 to 2020. We observed that in all annual periods from April to September (including half of a year) between 2011 and 2020, only 8 cases occurred, accounting for 14.03% of all cases. To further analyze the correlation between the onset of delirium and the local climate, cases that occurred in different months of the year were correlated with the average maximum and minimum temperatures. We observed that in months with higher temperatures (especially with an average minimum temperature >20°C), the incidence of delirium was much lower than that in other months. Altogether, very few cases were observed in local summer (1, 0, and 1 case for June, July, and August, respectively), accounting for 3.51% of all cases.

### 3.3. Prognosis analyses for patients with different durations to recovery

A comparison of the prognoses between the patients in early and delayed recovery groups is listed in Table 1. Fewer cases of death (5.56% vs 57.14%, *P* = .000) and lower hospitalization costs (7968.42 ± 3438.43 CNY vs 12852.38 ± 9361.13 CNY, *P* = .031) were observed in patients with early recovery than in those with delayed recovery.

**Table 1:** Prognoses for patients with different recovery.

	Total (n = 57)	Early recovery (n = 36)	Delayed recovery (n = 21)	*P* value
Death, n (%)	14 (24.56)	2 (5.56)	12 (57.14)	<.001*
Readmission, n (%)	47 (82.46)	27(75.00)	20 (95.24)	.074
Cost for hospitalization, CNY	9767.77 ± 6658.48	7968.42 ± 3438.43	12,852.38 ± 9361.13	.031*

* *P* < .05.

**Figure 2.**
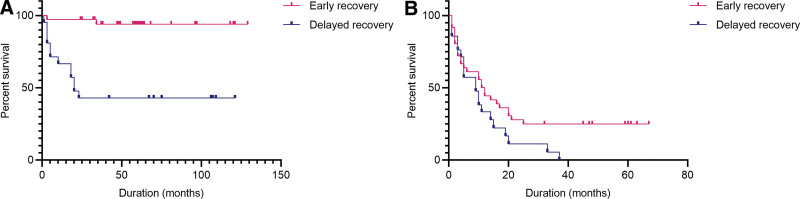
Kaplan–Meier curves for occurrence of death (A) and readmission (B) in patients with different recovery.

For further analysis, a Kaplan–Meier survival curve analysis was performed to compare death and readmission rates for patients with different durations to recovery. As shown in Figure 2, lower mortality was observed in patients with early recovery than in those with delayed recovery (*P* = .000, hazard ratio [HR] = 0.066, 95% confidence interval [95% CI] = 0.021–0.212). A lower readmission trend was observed in patients with early recovery than in those with delayed recovery, but this was not statistically significant (*P* = .292, HR = 0.550, 95% CI = 0.287–1.051).

### 3.4. Independent factors for delayed recovery

For further comparison of factors influencing recovery, a propensity score matching (1:1) was conducted; 19 cases were discarded, leaving 38 cases for logistic regression analyses. To determine the influential factors for recovery, univariate logistic regression analysis for delayed recovery was applied to screen the collected factors. Three variables, including insomnia, urine volume, and dialysis age, were selected for statistical significance (Table S2, Supplemental Digital Content, http://links.lww.com/MD/J165).

**Figure 3.**
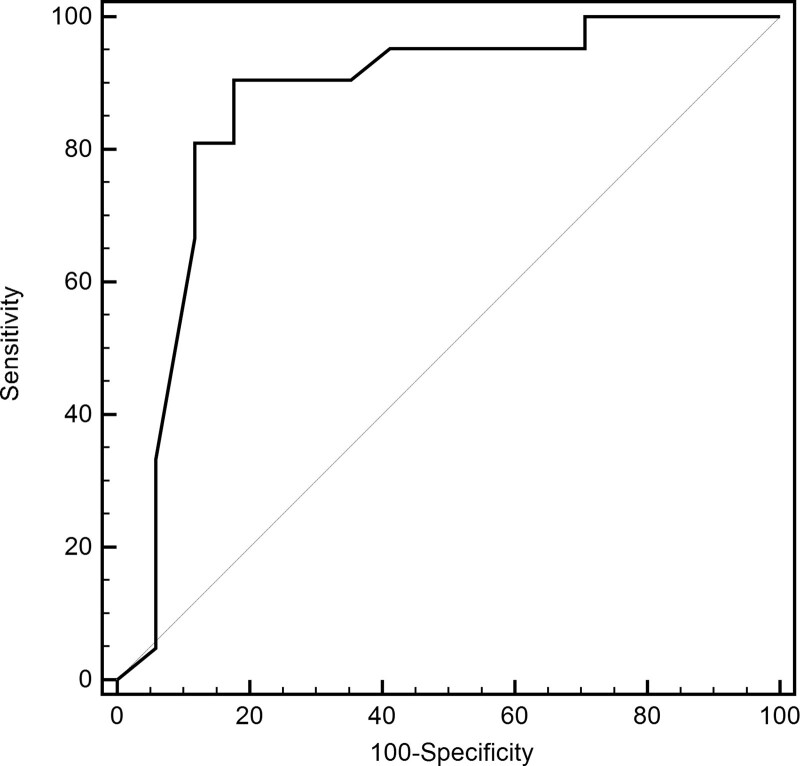
ROC curve for the model of the multivariate logistic regression. ROC = receiver operator characteristic.

To find the independent factors, the screened factors mentioned above were included in a multivariate binary logistic regression analysis (with the method of “Forward: LR”). Insomnia (*P* = .032, $\hat{OR}$ = 6.844, 95% CI = 1.179–39.711) and urine volume > 300 mL (*P* = .014, $\hat{OR}$ = 0.054, 95% CI = 0.005–0.599) were selected as an independent risk and protective factor for poorer recovery, respectively (Table 2). Considering that the cumulative amantadine dose can be closely related to recovery, adjusted multivariate binary logistic regression analysis (with the method of “Enter”) was conducted. As a result, similar trends remained for insomnia (*P* = .022, $\hat{OR}$ = 10.119, 95% CI = 1.403–72.900) and volume > 300 mL (*P* = .029, $\hat{OR}$ = 0.018, 95% CI = 0.006–0.621), whereas increment (per 100 mg) of cumulative dose (*P* = .190, $\hat{OR}$ = 1.588, 95% CI = 0.395–3.172) tended to be a risk of delayed recovery. The area under curve of the receiver operating characteristic curve was 0.867, with a sensitivity of 90.5% and a specificity of 82.4% at the cutoff point (cutoff = 0.432, Fig. 3).

**Table 2:** Multivariate logistic regression analysis for delayed recovery.

Variables	b	SE (b)	Wald	*P* value	$\hat{OR}$	OR 95% CI
Forward: LR						
Insomnia (AIS score > 10)	1.923	0.897	4.597	.032*	6.844	1.179–39.711
Urine volume > 300 mL	−2.913	1.189	5.997	.014*	0.054	0.005–0.599
Enter						
Insomnia (AIS score > 10)	2.314	1.008	5.270	.022*	10.119	1.403–72.990
Urine volume > 300 mL	−2.790	1.180	5.587	.029*	0.018	0.006–0.621
Cumulated dose of amantadine (per 100 mg)	0.462	0.353	1.716	.190	1.588	0.795–3.172

AIS = Athens insomnia scale, OR = odd ratio, SE = standard error.

* *P* < .05.

## 4. Discussion

In this study, more male patients had amantadine-associated delirium than the female patients did. This could be explained by the higher percentage of male patients treated with hemodialysis observed in the international Dialysis Outcomes and Practice Patterns Study.^[20]^ The contribution of primary cause of ESRD generally conformed to the national contribution reported previously,^[21]^ which presented that delirium has no specific susceptibility to the primary cause.

An uneven seasonal distribution was observed in the occurrence of amantadine-associated delirium in dialysis patients. A similar trend was found in a previous study on delirium in hospitalized patients,^[22]^ which revealed the occurrence of more cases in winter. This could be related to the epidemiological distribution of pulmonary diseases with an increasing probability of improper drug administration. In another study involving intensive care unit patients, occurrences among different seasons exhibited no significant variance.^[23]^ In this study of patients undergoing maintenance dialysis in a local hospital, the seasonal variance tended to be more noticeable, with fewer cases in months with a minimum temperature >20°C. To explain this phenomenon, perspiration, an amplified way of excretion in dialysis patients, should be taken into consideration. Although the excretion of amantadine in sweat has rarely been investigated, a similar excretion type of glandular secretion through latex and saliva has already been proven,^[24]^ which would help explain the missing part of amantadine excretion other than through the kidneys (approximately 90%) in healthy adults.^[24]^ Meanwhile, the compensation for sweat excretion in dialysis has also been pervasively observed in different studies. Increased perspiration in the summer can help maintain a balance between water and electrolytes via warm temperatures.^[25–27]^ Significant differences in waste excretion have been reported between maintenance hemodialysis patients and their healthy controls.^[28]^ The above clues indicated that a higher temperature with more perspiration could be a potential sign of reduction in the incidence of amantadine-associated delirium in dialysis patients.

Regarding recovery and prognosis, the survival and hospitalization costs for patients with early recovery (recovered within 14 days) were statistically superior to that for patients with delayed recovery. Our results coincided with previous studies showing that prolonged duration of delirium can cause poorer prognoses in dialysis patients.^[6,29–31]^ However, a recent study involving intensive care unit patients^[32]^ showed no significant difference in cognitive prognosis between different durations of recovery from metabolic delirium in patients with acute impairment of kidney or liver function. This may be due to different definitions of early recovery and the dramatic interference with long-term prognosis caused by high mortality in patients with multiple organ dysfunction syndromes.

Regarding the factors influencing the outcome of delayed recovery, insomnia was observed as an independent risk factor, and urine volume > 300 mL was observed as a protective factor for poorer recovery. It was not surprising that urine volume, an observable index of residual renal function, was selected as a protective factor, as amantadine excretion largely relies on kidney function rather than dialysis.^[11]^ This may also explain the differences between patients with early and delayed recovery attributed to dialysis age. However, it is worth examining how insomnia affects the duration of recovery. Sleep-related variables are closely related to different types of mental disorders.^[33]^ Furthermore, it has been revealed recently that sleep is a crucial way of waste clearance in the brain associated with the cerebrospinal fluid flow,^[34]^ suggesting that sleep may play an important role in amantadine excretion by weakening its effects on the brain.^[35]^ On the contrary, insomnia causes loss of this function, resulting in delayed recovery. More importantly, although insomnia can be relieved by medication,^[36]^ this is not always a priority, whereas urine volume cannot be increased for most patients on maintenance dialysis. The importance of sleep improvement revealed in this study may provide a strategy for the treatment of amantadine-associated delirium in dialysis patients.

However, there were certain limitations to this study. First, there were difficulties in targeting all patients administered amantadine before the occurrence of delirium due to the various approaches for drug purchase. Second, some patients with mild symptoms recovered without hospitalization or even without a hospital visit. The failure to include these patients might have caused a statistical bias. Studies involving multiple centers are necessary for more concrete data.

## 5. Conclusions

In patients undergoing maintenance dialysis, amantadine-associated delirium exhibited uneven seasonal distribution, with few cases in local summer. Patients with early recovery had better prognoses that those with delayed recovery. Sleeping status and urine volume are important factors for recovery, suggesting that early recovery should be the aim of treatment by prioritizing insomnia treatment. Higher temperatures could be a potential sign of reduction in the incidence of amantadine-associated delirium in dialysis patients.

## Author contributions

**Conceptualization:** Jing Li, Jianqiang He.

**Data curation:** Jing Li, Bolin Si, Jun Chao, Jianqiang He.

**Formal analysis:** Jing Li, Bolin Si, Jun Chao, Jianqiang He.

**Investigation:** Jing Li.

**Methodology:** Jing Li, Jianqiang He.

**Project administration:** Jianqiang He.

**Resources:** Jing Li.

**Visualization:** Jianqiang He.

**Writing – original draft:** Jing Li.

**Writing – review & editing:** Jing Li, Bolin Si, Jun Chao, Jianqiang He.

## Supplementary Material
