# Supplementary material for: Amantadine-associated delirium in patients with maintenance dialysis: Insomnia-associated recovery and uneven seasonal distribution
Source: Medicine (Baltimore). 2023 Jun 30;102(26):e34077. doi: 10.1097/MD.0000000000034077 (PMC10313273; doi:10.1097/MD.0000000000034077)
Supplement: Supplementary file 1 [file medi-102-e34077-s001.pdf]

Table A: Clinical characteristics of amantadine-associated delirium in patients with maintenance dialysis and comparisons for different recovery.

**Variables**	**Total (*n*=57)**	**Early recovery (*n*=36)**	**Delayed recovery (*n*=21)**	***P* value**
**Demographics and dialysis**				
Male, n (%)	38 (66.67)	22(61.11)	16 (76.19)	0.244
Age, years	63.54 ± 12.35	61.72 ± 12.40	66.67 ± 11.92	0.146
Urine volume > 300ml, n (%)	18(31.58)	17 (47.22)	1 (4.76)	0.001**
*Hemodialysis, n (%)	49 (85.96)	32 (88.89)	17 (80.95)	0.449
Dialysis age, years	2.43 ± 1.84	2.32 ± 1.95	2.61 ± 1.68	0.560
Dialysis age < 1 year, n (%)	22 (38.60)	18 (50.00)	4 (19.05)	0.021**
**History and comorbid conditions**				
Insomnia, n (%)	32 (56.14)	14 (38.89)	18 (85.71)	0.001**
Antibiotic administration, n (%)	28 (49.12)	19 (52.77)	9(42.86)	0.470
Cumulated dose of amantadine (per 100mg)	7.16 ± 1.36	7.00 ±1.33	7.42 ± 1.40	0.255
Duration of amantadine administration	5.08 ± 1.23	5.13 ± 1.22	5.00 ± 1.26	0.648
*Hypertension, n (%)	47 (82.46)	30 (83.33)	17(80.95)	1.000
Diabetes mellitus, n (%)	23 (40.35)	15 (41.67)	8(38.10)	0.791
*Cerebrovascular disease, n (%)	7(12.28)	6(16.67)	1(4.76)	0.243
*Heart diseases, n (%)	9 (15.79)	7 (19.44)	2 (9.52)	0.461
*COPD, n (%)	10 (17.54)	6 (16.67)	4 (19.05)	1.000
*Hepatitis, n (%)	10 (17.54)	4 (11.11)	6 (28.57)	0.148
**Laboratory tests at admission**				
Serum creatine (pre-dialysis), umol/L	835.27 ± 314.37	826.21 ± 342.62	850.79 ± 266.26	0.779
Blood urea nitrogen (pre-dialysis), umol/L	19.32 ± 7.31	20.72 ± 8.06	16.93 ± 5.15	0.059
Hemoglobin, g/L	96.19 ± 19.14	94.50 ± 21.38	99.47± 16.66	0.543

Alanine aminotransferase, U/L	16.46 ± 20.69	17.98 ± 25.28	13.85 ± 8.31	0.474
Aspartate aminotransferase, U/L	22.01 ± 15.74	20.86 ± 16.59	23.99 ± 14.35	0.360
Albumin, g/L	36.14 ± 5.67	36.14± 5.06	35.35 ± 6.60	0.614
Total bilirubin, umol/L	6.16 ± 3.22	5.90 ± 3.24	6.61 ± 3.22	0.426
Parathyroid hormone, pg/mL	426.34 ± 327.26	422.65 ± 282.60	432.66 ± 400.07	0.912
Serum phosphate, mmol/L	1.70 ± 0.38	1.70 ± 0.39	1.70 ± 0.36	0.986
C-reactive protein, mg/L	19.91 ± 23.81	18.31 ± 19.68	22.65 ± 29.94	0.512
Triglycerides, mmol/L	1.52 ± 0.61	1.61 ± 0.67	1.37 ± 0.47	0.168
Total Cholesterol, mmol/L	3.85 ± 0.90	4.02 ± 0.88	3.56 ± 0.89	0.063
**Treatments**				
*Hemoperfusion, n (%)	50 (87.72)	33 (91.67)	17 (80.95)	0.404
Olanzapine, n (%)	35 (61.40)	20 (55.56)	15 (71.43)	0.235
*Quetiapine, n (%)	10 (17.54)	7 (19.44)	3 (14.29)	0.730
*Haloperidol, n (%)	12 (21.05)	6 (16.67)	6 (28.57)	0.327

* *P* value calculated with *Fisher's* exact test.

**P < 0.05.
