# Supplementary material for: Amantadine-associated delirium in patients with maintenance dialysis: Insomnia-associated recovery and uneven seasonal distribution
Source: Medicine (Baltimore). 2023 Jun 30;102(26):e34077. doi: 10.1097/MD.0000000000034077 (PMC10313273; doi:10.1097/MD.0000000000034077)
Supplement: Supplementary file 2 [file medi-102-e34077-s002.pdf]

Table B: Significant and amantadine-related variables in the univariate logistic regression analysis for delayed recovery.

**Variables**	***b***	***SE(b)***	***Wald***	***P* value**	$\widehat{OR}$	***OR* 95% *CI***
Dialysis age < 1 year	-1.565	0.738	4.493	0.034*	0.209	0.049-0.889
Insomnia (AIS score >10)	2.148	0.795	7.306	0.007*	8.571	1.805-40.701
Urine volume>300mL	-3.114	1.134	7.537	0.006*	0.044	0.005-0.410
Cumulated dose of amantadine (per 100mg)	0.149	0.228	0.428	0.513	1.161	0.742-1.817
Duration of amantadine administration	-0.090	0.285	0.099	0.753	0.914	0.523-1.598

*P < 0.05.
